# ERRα inhibitor acts as a potential agonist of PPARγ to induce cell apoptosis and inhibit cell proliferation in endometrial cancer

**DOI:** 10.18632/aging.104049

**Published:** 2020-11-10

**Authors:** Meimei Huang, Lili Chen, Xiaodan Mao, Guifen Liu, Yuqin Gao, Xiaoqing You, Min Gao, Jalid Sehouli, Pengming Sun

**Affiliations:** 1 Department of Gynecology and Obstetrics, Fujian Maternity and Child Health Hospital, Affiliated Hospital of Fujian Medical University, Fuzhou 350001, China; 2 Laboratory of Gynecologic Oncology, Fujian Maternity and Child Health Hospital, Affiliated Hospital of Fujian Medical University, Fuzhou 350001, China; 3 Reproductive Center, Fujian Maternity and Child Health Hospital, Affiliated Hospital of Fujian Medical University, Fuzhou 350001, Fujian, P.R. of China; 4 Department of Cell Biology and Genetics, School of Basic Medical Sciences, Fujian Medical University, Fuzhou 350108, Fujian, China; 5 Department of Gynecologic Oncology, Peking University Cancer Hospital, Beijing 100046, China; 6 Department of Gynecology, Campus Virchow Clinic, CharitéUniversitätsmedizin Berlin, Humboldt University of Berlin, Berlin 13353, Germany

**Keywords:** peroxisome proliferator-activated receptor-γ, estrogen-related receptor-α, endometrial cancer, carcinogenesis, prognosis

## Abstract

Two transcriptional factors, peroxisome proliferator-activated receptor-γ (PPARγ) and estrogen-related receptor-α (ERRα), have been reported to be key regulators of cellular energy metabolism. However, the relationship between ERRα and PPARγ in the development of endometrial cancer (EC) is still unclear. The expression levels of PPARγ and ERRα in EC were evaluated by quantitative real-time PCR, western blot, tissue array and immunohistochemistry. A significant negative correlation was identified between PPARγ and ERRα expression in women with EC (ρ=-0.509, P<0.001). Bioinformatics analyses showed that PPARγ and ERRα can activate or inhibit the same genes involved in cell proliferation and apoptosis through a similar ModFit. ERRα activation or PPARγ inhibition could promote proliferation and inhibit apoptosis through the Bcl-2/Caspase3 pathways. Both PPARγ and ERRα can serve as serum tumor markers. Surprisingly, as evaluated by receiver operating characteristic (ROC) curves and a logistic model, a PPARγ/ERRα ratio≤1.86 (area under the ROC curve (AUC)=0.915, Youden index=0.6633, P<0.001) was an independent risk factor for endometrial carcinogenesis (OR=14.847, 95% CI= 1.6-137.748, P=0.018). EC patients with PPARγ(-)/ERRα(+) had the worst overall survival and disease-free survival rates (both P<0.001). Thus, a dynamic imbalance between PPARγ and ERRα leads to endometrial carcinogenesis and predicts the EC prognosis.

## INTRODUCTION

Endometrial cancer (EC) is the second most common gynecological tumor [1, 2]. In 2019, approximately 61,880 new EC cases and 12,160 deaths due to EC were reported in the United States [1]. Recently, the incidence of EC has markedly increased in China, likely due to the growing obesity epidemic as obesity is a strong risk factor for EC [3]. Although most ECs can be found in the early stage, the prognosis of patients with high-grade, poorly differentiated, advanced-stage EC is poor. The use of traditional tumor biomarkers, namely, CA125, CA199, CA153 and CEA, to monitor EC has produced unsatisfactory results in clinical practice [4, 5]. Therefore, tumor biomarkers for predicting and monitoring EC are still needed.

Peroxisome proliferator-activated receptor γ (PPARγ), a steroid hormone receptor, was discovered by Issemann and Green in 1990 and plays an important role in the regulation of glucose and lipid metabolism in some obesity-related cancers [6], such as breast cancer [7] and EC [8]. Early in 2006, Kyoko O et al. suggested that PPARγ immunoreactivity and mRNA levels were significantly lower in EC than in the normal endometrium [9]. Nickkho-Amiry et al. found that PPARγ activation reduced endometrial cell proliferation, that nuclear PPARγ was most abundant in benign endometrial tissue, and that nuclear PPARγ correspondingly decreased with increasing pathological grades [8]. Thus, PPARγ seems to act as a protective factor against EC. However, other studies have shown that PPARγ has protumorigenic effects in pancreatic cancer [10], metastatic prostate cancer [11], and bladder carcinoma [12]. Thus, the role of PPARγ in cancer remains controversial. Wei W et al. identified a conserved PPAR response element (PPRE) in the estrogen-related receptor α (ERRα) promoter and suggested that ERRα was a direct PPARγ target gene, specifically in osteoclastogenesis, by ChIP assay [13]. Coincidentally, Fujimoto J et al. demonstrated that ERRα was expressed at higher levels in EC tissues and was correlated with a poor prognosis in EC [14]. Moreover, we found that ERRα reduced EC cell proliferation and promoted apoptosis [15–17]. Thus, ERRα is a potential tumor biomarker and therapeutic target for EC. However, the potential mechanisms of ERRα and PPARγ in tumorigenesis are still unclear. In PubMed, no report focusing on the interaction between ERRα and PPARγ in EC is available. Therefore, we hypothesized that some crosstalk might occur between PPARγ and ERRα to promote EC carcinogenesis and progression. To clarify the possible biological and clinical roles of PPARγ and/or ERRα in human EC, we focused on the interaction between PPARγ and ERRα in EC cells *in vitro* and EC tissues *in vivo*. Furthermore, the downstream target pathways of PPARγ and ERRα in EC were explored based on bioinformatics data-mining analysis and further confirmed in cell lines. We also discuss the potential roles of PPARγ and ERRα in the EC diagnosis and try to determine the best threshold. Finally, the disease-free survival (DFS) and overall survival (OS) rates of EC patients with different expression patterns of PPARγ and ERRα were analyzed.

## RESULTS

### PPARγ is negatively correlated with ERRα in EC tissue

**Figure 1:**
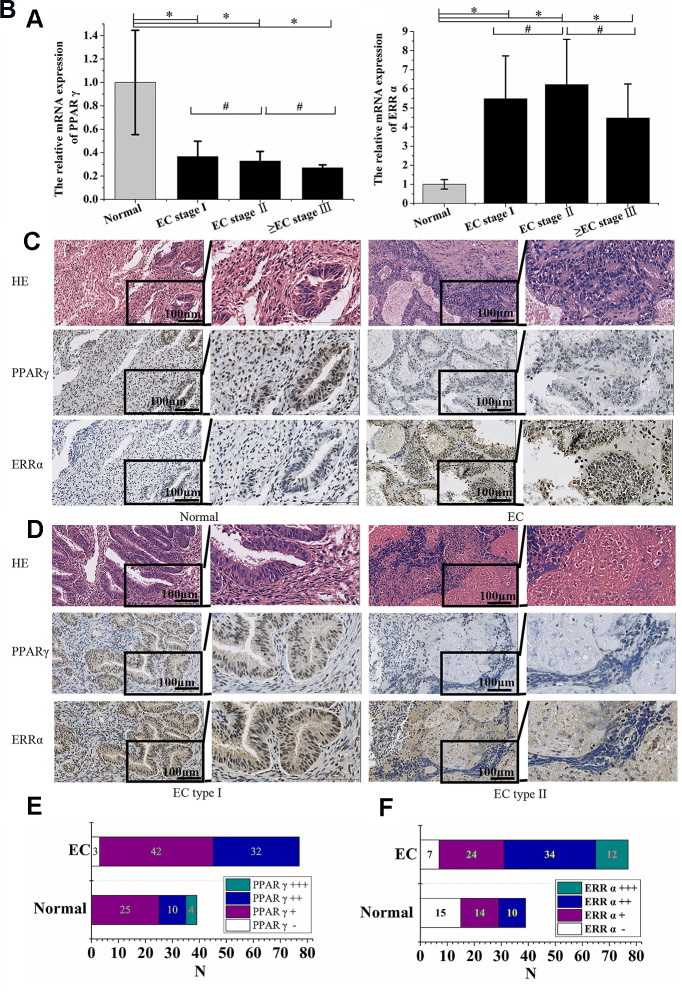
**PPARγ was negatively correlated with ERRα in EC tissue.** (**A**) The relative mRNA expression of PPARγ and (**B**) the relative mRNA expression of ERRα in EC patients with different FIGO stages. (**C**) Immunohistochemical expression of PPARγ and ERRα in the normal endometrium and EC (×200 & ×400). (**D**) Immunohistochemical expression of PPARγ and ERRα in EC type I and EC type II (×200 & ×400). (**E**) Immunohistochemical expression of PPARγ in the normal endometrium and EC. (**F**) Immunohistochemical expression of ERRα in the normal endometrium and EC. CON: normal endometrium. EC, endometrial cancer. *, P<0.05; #, P>0.05.

The expression of PPARγ mRNA in 77 EC patient tissues and 39 normal control tissues was determined by qRT-PCR. Compared to normal endometrium tissues, lower expression of PPARγ in EC tissues was noted (P<0.05, Figure 1A). In contrast, EC tissue showed higher expression of ERRα (P<0.05, Figure 1B). No significant differences in either PPARγ or ERRα expression were found among EC patients with different FIGO stages (P>0.05, Figure 1A, 1B). PPARγ and ERRα protein expression was also detected by immunohistochemistry. Positive immunoreactivity for PPARγ was detected in the nuclei of carcinoma cells and normal endometrial gland cells. Immunoreactivity of PPARγ was significantly lower in endometrial carcinoma tissue than in normal endometrial tissue (P<0.001, Figure 1C, 1E). However, the ERRα levels detected in 70 of 77 (90.1%) EC tissue samples were higher than those detected in 24 of 39 (61.54%) normal endometrial tissue samples (P<0.001, Figure 1C, 1F). Thus, PPARγ and ERRα presented opposing expression patterns in both normal endometrial and EC tissues. PPARγ immunoreactivity was lower in type II EC than in type I EC (P<0.05, Figure 1D), but no obvious difference in ERRα immunoreactivity was observed between the two types of EC (P>0.05, Figure 1D). The correlations between the expression of PPARγ and ERRα and patient clinicopathologic variables, including stage and pathological type and grade, were summarized in Table 1. Based on the data, a negative correlation was identified between PPARγ immunoreactivity and ERRα immunoreactivity according to Spearman’s rank correlation analysis (ρ=-0.509, P<0.001).

**Table 1:** Correlation between PPARγ, ERRα expression immunoreactivity in EC.

	**N0.of cases**	**PPARγ-positive**	**%**	**P^a^**	**ERRα-positive**	**%**	**P^a^**
FIGO stage							
I	48	40	83.33		41	85.42	
II-IV	29	14	48.28	0.001	29	1	0.08
Type							
I	63	48	76.19		56	88.89	
II	14	6	42.86	0.014	14	1	0.427
Grade							
G1	27	23	85.19		24	88.89	
G2	29	23	79.31		27	93.1	
G3	7	2	28.57	0.014	5	71.43	0.276

^a^The results were evaluated using χ-square test.

### Functional annotation and pathway enrichment for PPARγ and ERRα

**Figure 2:**
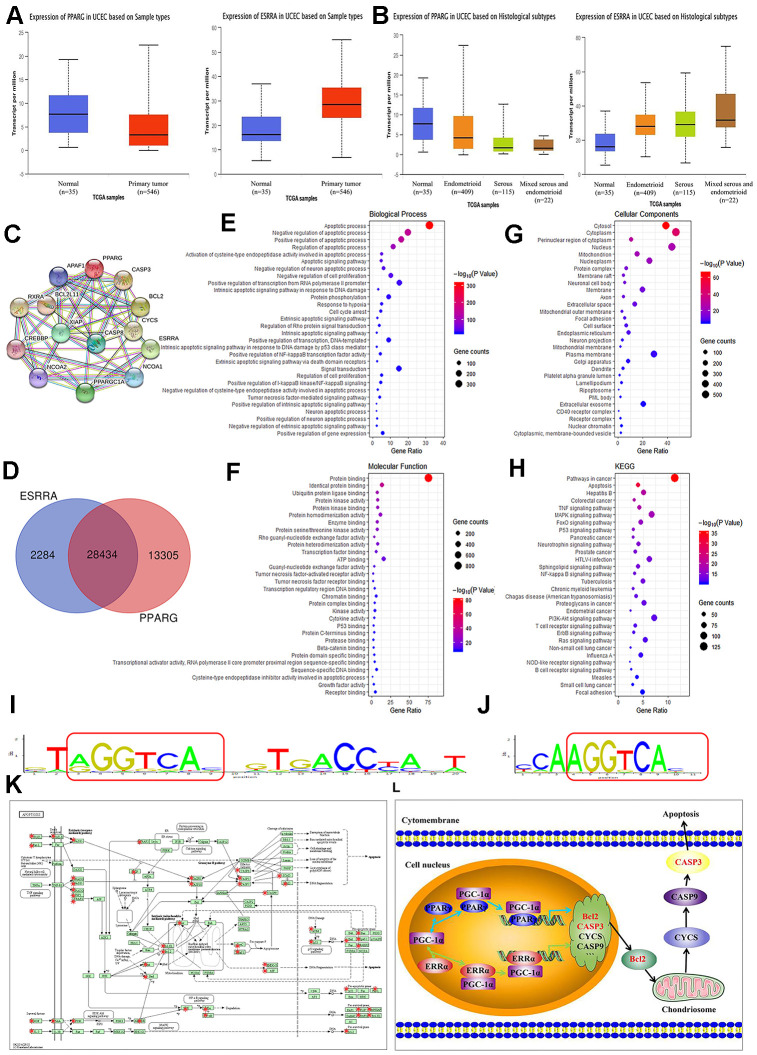
**Summary of functional annotation and pathway enrichment for PPARγ and ERRα.** (**A**) Boxplot showing the relative expression of PPARγ and ERRα in normal and EC samples from the UALCAN database. (**B**) Boxplot showing the relative expression of PPARγ and ERRα in EC based on histological subtypes from the UALCAN database. (**C**) Protein mapping for PPARγ and ERRα based on string data. (**D**) Venn diagram showing the common target genes of PPARγ and ERRα. (**E**–**G**) GO enrichment of the common target genes of PPARγ and ERRα. (**H**) KEGG enrichment of the target genes regulated by PPARγ and ERRα. (**I**) The ModFit of PPARγ based on the JASPAR database. (**J**) The ModFit of ERRα based on the JASPAR database. (**K**) KEGG pathway maps for apoptosis; The red dots represent target genes of PPARγ and ERRα. (**L**) Possible mechanism of interaction between PPARγ and ERRα. EC, endometrial cancer. *, P<0.05; #, P>0.05.

PPARγ and ERRα gene expression and EC were selected and queried on the http://ualcan.path.uab.edu/ website. UALCAN lists queried genes with links to gene expression analyses and survival data. Analysis of TCGA samples showed that PPARγ expression was higher in normal samples (n=35), and that ERRα expression was obviously increased in EC samples (n=546, P<0.001, Figure 2A). Moreover, PPARγ expression was higher in endometrioid adenocarcinoma (n=409) than in serous carcinoma (n=115, P<0.001, Figure 2B). However, no difference in ERRα expression was found between endometrioid adenocarcinoma and serous carcinoma (P>0.05, Figure 2B). These data were all consistent with our results described above. Although the STRING analysis revealed no research overlap between PPARγ and ERRα, the same cofactors, namely, PPARγ coactivator-1α (PGC-1α), nuclear receptor coactivator 1 (NCOA1), and cyclic adenosine monophosphate response element-binding protein (CREBBP), were reported to be shared between them. CREBBP can transcribe, coactivate and increase the expression of its target genes. PPARγ and ERRα can compete with the cofactor CBP to increase or decrease Bcl2 expression (Figure 2C). As shown in Figure 2D, the microarray data revealed 41,739 target genes of PPARγ and 30,718 target genes of ERRα. A total of 28,434 identical target genes, including Bcl2 and Caspase3, were found between PPARγ and ERRα (Figure 2D). Figure 2E–2G graphically depicts the most common GO codes for the three major GO categories. Further searches revealed a repeating sequence in both the PPARγ and ERRα genes (Figure 2I, 2J). These data suggested that a significant number of protein-coding genes with important biological functions, especially in the apoptosis pathway, may be regulated by PPARγ and ERRα. By KEGG analysis, we found that a large number of genes coregulated by PPARγ and ERRα are involved in the apoptosis pathway (Figure 2K). Further confirming our hypothesis, PPARγ and ERRα compete for the same cofactors and increase or decrease the same genes, including Bcl2 and Caspase3, involved in the apoptosis pathway (Figure 2L).

### Reciprocal inhibition between ERRα and PPARγ in EC cells

**Figure 3:**
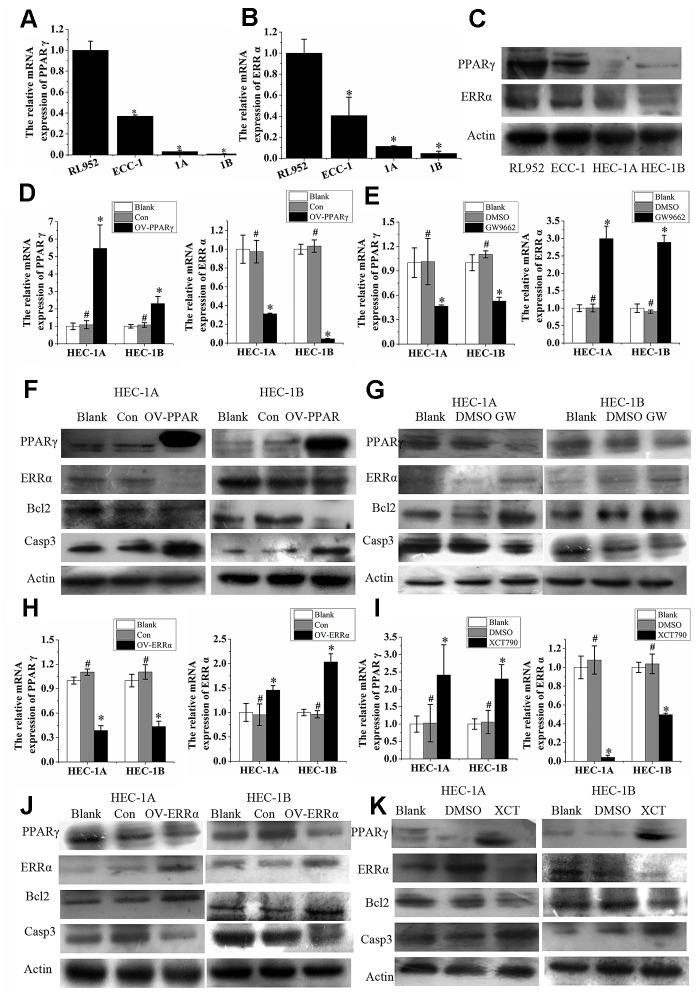
**ERRα and PPAR γ negatively regulate each other in EC cells.** (**A**) The relative mRNA expression of PPARγ and (**B**) the relative mRNA expression of ERRα in RL952, ECC-1, HEC-1A, and HEC-1B cells. (**C**) Protein expression of PPAR γ and ERRα in RL952, ECC-1, HEC-1A, and HEC-1B cells. (**D**) Upregulated PPARγ and (**E**) suppressed PPARγ by GW9662: the relative mRNA expression of PPARγ and ERRα in EC cells. (**F**) Upregulated PPARγ and (**G**) suppressed PPARγ by GW9662: the protein expression of PPARγ and ERRα in EC cells. (**H**) Upregulated ERRα and (I) suppressed ERRα by XCT790: the relative mRNA expression of ERRα and PPARγ in EC cells. (**J**) Upregulated ERRα and (**K**) suppressed ERRα by XCT790: the protein expression of PPAR γ and ERRα in EC cells. OV-PPARγ, overexpression of PPARγ. OV-ERRα, overexpression of ERRα. EC, endometrial cancer. *, P<0.05; #, P>0.05.

PPARγ and ERRα were both expressed in EC cell lines, including RL952, ECC-1, HEC-1A, and HEC-1B. Interestingly, the mRNA expression of PPARγ and ERRα was higher in the ERα-positive cell lines RL952 and ECC-1 than in the ERα-negative cells HEC-1A and HEC-1B (P<0.05, Figure 3A, 3B). The proteins were also expressed at high levels in the ERα-positive lines RL952 and ECC-1 and at low levels in the ERα-negative lines HEC-1A and HEC-1B (P<0.05, Figure 3C). A similar tendency was observed for PPARγ and ERRα protein expression in these EC cells. The semiquantitative PPARγ protein expression levels in RL952, ECC-1 HEC-1A, and HEC-1B cells were 1±0.0611, 0.6313±0.0696, 0.1566±0.0069, and 0.2027±0.0274, respectively, and the semiquantitative ERRα protein expression levels in RL952, ECC-1 HEC-1A, and HEC-1B cells were 1±0.0699, 1.0429±0.0378, 0.7135±0.0374, and 0.6514±0.0948, respectively. PPARγ expression was significantly increased, while ERRα expression was significantly reduced by lentivirus-mediated PPARγ overexpression in HEC-1A and HEC-1B cells (Figure 3D). PPARγ expression was decreased, while ERRα expression was increased when the same cells were treated with GW9662 (Figure 3E), a potent antagonist of PPARγ [18]. When PPARγ was overexpressed, Bcl2 protein expression was decreased, and Caspase3 protein expression was increased (Figure 3F). After treatment with G9662 for 48 h, Bcl2 protein expression was increased, and Caspase3 protein expression was decreased (Figure 3G). When ERRα was overexpressed in cells, PPARγ was downregulated (Figure 3H). Subsequently, ERRα was upregulated, and PPARγ was downregulated when ERRα was overexpressed by lentivirus in HEC-1A and HEC-1B cells (Figure 3H). ERRα was decreased, but PPARγ was increased in the same cells after treatment with XCT790, a specific ERRα antagonist (Figure 3I). When ERRα was upregulated, Bcl2 protein expression was increased, and Caspase3 protein expression was decreased (Figure 3J). After treatment with XCT790 for 48 h, Bcl2 protein expression was decreased, and Caspase3 protein expression was increased (Figure 3K). Taken together, these data indicate that PPARγ and ERRα participate in a negative feedback loop in EC. This regulatory pattern was also true for the Bcl2 and Caspase3 proteins (Figure 3F–3G and Figure 3J–3K).

### PPARγ and ERRα compete to activate or inhibit cell proliferation and apoptosis through Bcl2/Caspase3 signals

**Figure 4:**
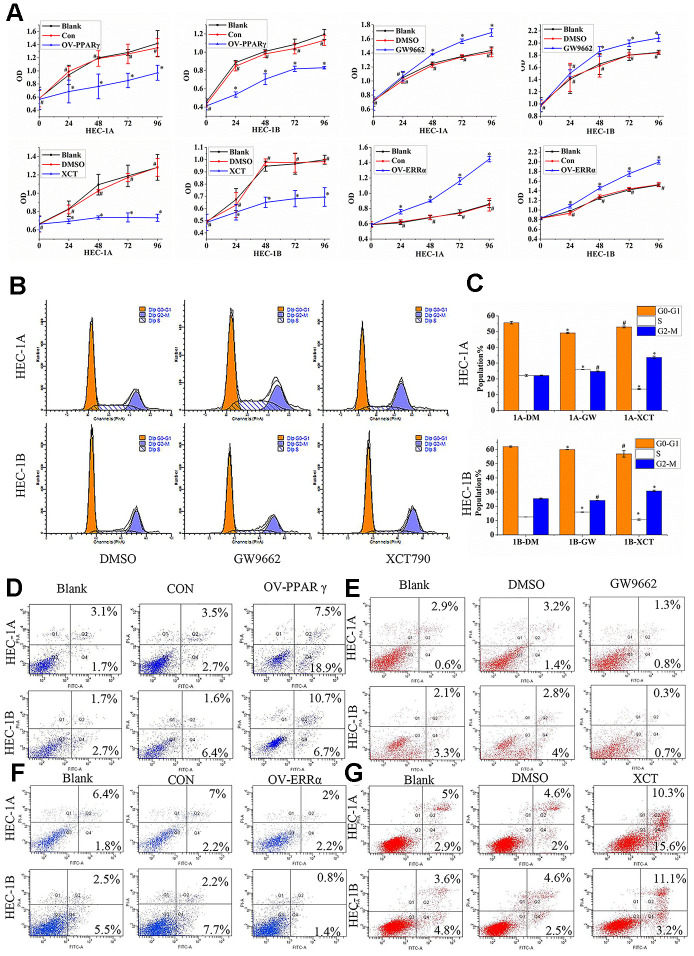
**PPARγ and ERRα compete to control cell proliferation and promote apoptosis in EC cells.** (**A**) The effect of OV-PPARγ, OV-ERRα, GW9662 or XCT790 on proliferation. The OD values of HCE-1A and HEC-1B cells were detected at 0, 24, 48, 72, and 96 h after transfection with lentivirus or treatment with ERRα and PPARγ antagonists. (**B**, **C**) The effect of GW9662 or XCT790 on the cell cycle. HEC-1A and HEC-1B cells were treated with DMSO, GW9662 (5 μM) or XCT790 (10 μM) for 72 h. (**D**, **E**) The effect of OV-PPARγ or GW9662 on apoptosis.(**F**, **G**) The effect of OV-ERRα or XCT790 on apoptosis. OV-PPARγ: overexpression of PPARγ; OV-ERRα: overexpression of ERRα. EC, endometrial cancer. *, P<0.05; #, P>0.05.

Our results showed that PPARγ overexpression slowed proliferation (Figure 4A), but that GW9662 treatment accelerated the proliferation of HEC-1A and HEC-1B cells (Figure 4A). Moreover, HEC-1A and HEC-1B cell proliferation was inhibited by XCT790 treatment (Figure 4A, P<0.05). The cellular proliferation rate was significantly increased when ERRα was overexpressed by a lentivirus (Figure 4A, P<0.05). The HEC-1A and HEC-1B cell cycles were analyzed after PPARγ and ERRα modulation. The data suggested that PPARγ downregulation by GW9662 led to G_0_-G_1_ phase shortening and S phase lengthening, while ERRα downregulation by XCT790 resulted in S phase shortening but G_2_-M phase lengthening in HEC-1A cells (Figure 4B-C). The same trend for the S phase and M phase was found in HEC-1B cells (Figure 4B, 4C). Apoptosis of HEC-1A cells and HEC-1B cells increased when PPARγ was upregulated (Figure 4D). In contrast, apoptosis of HEC-1A cells and HEC-1B cells decreased when PPARγ was downregulated (Figure 4E). Apoptosis of HEC-1A cells and HEC-1B cells decreased when ERRα was upregulated by a lentivirus (Figure 4F). Treatment with XCT790 significantly increased cell apoptosis (Figure 4G).

### The PPARγ/ERRα combination as a biomarker for EC diagnosis

**Figure 5:**
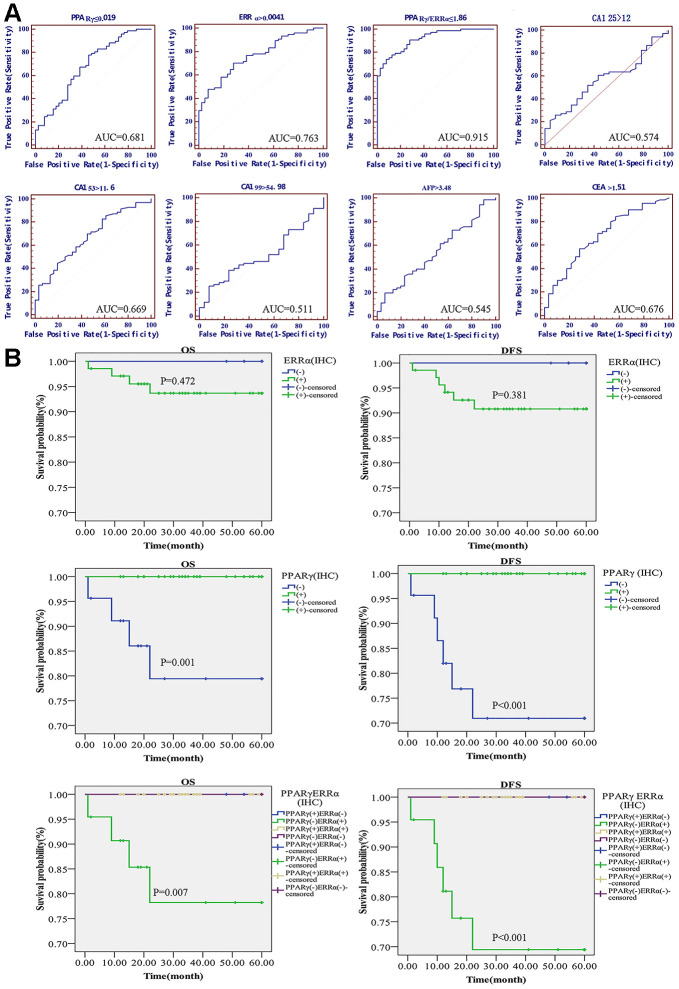
**Diagnostic and prognostic value of the PPARγ/ERRα ratio for EC.** (**A**) ROC curves of the mRNA expression of PPARγ, the mRNA expression of ERRα, the PPARγ/ERRα ratio, CA125, CA199, CA153, CEA, and AFP. (**B**) DFS and OS in EC patients with different expression patterns of PPARγ/ERRα. Patients with PPARγ(+)/ERRα(-) had longer DFS and OS. The mRNA expression of PPARγ and ERRα was quantified using the 2^-ΔCT^ method. PPARγ/ERRα was quantified as the ratio of the mRNA expression of PPARγ to the mRNA expression of ERRα. ROC, receiver operating characteristic; DFS, disease-free survival. OS, overall survival.

A receiver operating characteristic (ROC) curve was used to evaluate the diagnostic value of PPARγ and ERRα mRNA expression to further determine whether PPARγ and ERRα could serve as tumor markers. Classical serum biomarkers, including CA125, CA153, CA199, AFP and CEA, were also assessed in terms of their abilities to distinguish EC from benign lesions. At the highest area under the ROC curve (AUC, by best-point analysis), which was 0.681, PPARγ could distinguish women with benign lesions from those with EC tumors (Figure 5A). The AUC of ERRα was 0.763 (P<0.001) (Figure 5A). However, even though both AUCs were statistically significant, their sensitivity and specificity for predicting EC were poor. As a diagnostic predictor, ERRα seemed to be slightly better than PPARγ for EC. Surprisingly, when PPARγ and ERRα were combined to predict the genesis of EC, the AUC of PPARγ/ERRα was 0.915, with an optimal threshold of 1.86 (Youden index=0.6633, P<0.001); the predictive performance of this combination was substantially improved compared to that of each as a single indicator (Figure 5A). Other common clinical indicators, including CA125, CA153, CA199, AFP and CEA, were also analyzed by ROC curves (Figure 5A). The predictive abilities of these indicators were obviously lower than that of PPARγ/ERRα. We also determined whether PPARγ/ERRα could serve as an independent risk factor for EC. As shown in Table 2, we obtained meaningful indicators derived from clinical characteristics. After adjusting the data, we found that the risk of EC development in cases with PPARγ/ERRα ≤1.86 was higher than that in cases with PPARγ/ERRα >1.86 (OR = 14.847, 95% CI = 1.6-137.748, P=0.018).

**Table 2:** Logistic regression model: risk factors for endometrial cancer.

**Variables**	**OR**	**95%CI**	**P^a^**	**OR**	**95%CI**	**P^b^**
Age						
≤55	1(R)	-	0.001	1(R)	-	0.41
>55	33.366	4.358-255.432		4.49	0.126-160.285	
BMI						
<25	1(R)	-	0.004	1(R)	-	0.17
≥25	6.48	1.825-23.014		11.363	0.354-365.035	
Parity						
≤1	1(R)	-	0.002	1(R)	-	0.148
>1	3.777	1.642-8.688		0.22	0.028-1.712	
Gravidity						
≤2		-	<0.001		-	0.777
>2	13.667	3.877-48.181		1.571	0.069-35.596	
Pausimenia						
No	1(R)	-	<0.001	1(R)	-	0.237
Yes	11.1	3.149-39.124		4.703	0.362-61.067	
Hypertension						
No	1(R)	-	0.003	1(R)	-	0.573
Yes	9.99	2.234-44.677		2.93	0.07-122.652	
Hyperlipaemia						
No	1(R)	-	0.003	1(R)	-	0.47
Yes	9.99	2.234-44.677		0.218	0.003-13.557	
Diabetes mellitus						
No	1(R)	-	0.013	1(R)	-	0.789
Yes	13.333	1.717-103.56		0.603	0.015-24.505	
CA153						
≤31.3	1(R)	-	0.017	1(R)	-	0.835
>31.3	1.122	1.021-1.233		1.007	0.131-6.682	
CEA						
≤5	1(R)	-	0.02	1(R)	-	0.925
>5	2.055	1.12-3.771		0.955	0.944-1.073	
CA125						
≤35	1(R)	-	0.74	-	-	-
>35	1.001	0.997-1.005		-	-	
CA199						
≤37	1(R)	-	0.347	-	-	-
>37	1.004	0.996-1.012		-	-	
AFP						
≤8.78	1(R)	-	0.309	-	-	-
>8.78	1.237	0.821-1.864		-	-
PPARγ^c^						
>0.019	1(R)	-	0.001	1(R)	-	0.107
≤0.019	0.243	0.106-0.556		0.034	0.001-2.075	
ERRα^d^						
≤0.0041	1(R)	-	<0.001	1(R)	-	0.14
>0.0041	5.976	2.551-13.999		16.66	0.397-699.901	
PPARγ/ERRα^e^						
>1.86	1(R)	-	<0.001	1(R)	-	0.018
≤1.86	32	8.896-115.107		14.847	1.6-137.748	

^a^ The results were evaluated using univariate analysis.^b^The results were evaluated using logistic regression analysis. ^c^The mRNA expression of PPARγ was quantified using 2^-ΔCT^ method. ^d^ The mRNA expression of ERRα was quantified using 2^-ΔCT^ method. ^e^PPARγ/ERRα was quantified the ratio of mRNA expression of PPARγ and ERRα. FBG, fasting blood glucose.

### Prognostic value of the PPARγ/ERRα expression pattern

The 5-year OS rates were significantly higher for patients with PPARγ-positive disease than for those with PPARγ-negative disease (100.00% vs. 88.24%; p=0.001). The difference between the 5-year DFS rates for PPARγ-positive and PPARγ-negative patients was also significant (100.00% vs. 82.35%; P<0.001) (Figure 5B). However, no difference in OS or DFS was found between ERRα-positive and ERRα-negative patients. When using PPARγ/ERRα as a predictor of prognosis, we found that OS and DFS were lowest for PPARγ-negative and ERRα-positive patients (OS: 100.00% vs. 85.19%, P<0.001; DFS: 100.00% vs. 77.78%, P<0.001, Figure 5B).

## DISCUSSION

We found that the expression of PPARγ, whether at the transcriptional (mRNA) or posttranscriptional (protein) level, was reduced in EC, which is consistent with other reports [9, 19–23]. However, several studies also showed that PPARγ expression was more marked in carcinoma tissues of several types of human malignancies than in normal tissues [10–12]. PPARγ has been associated with clinicopathological variables, such as EC stage, which is in agreement with previous studies [19]. The results of our study are also consistent with these results, which support the hypothesis that the PPARγ gene is a tumor suppressor gene, and that dysfunction of PPARγ contributes to tumorigenesis [7, 24, 25]. In our present study, strong ERRα immunoreactivity was detected in endometrial carcinoma tissues. ERRα mRNA expression was also higher in carcinoma tissues than in normal tissues. Similar results have also been reported in other cancers [26–28]. No significant associations between ERRα and clinicopathological variables were identified, although the number of cases involved in our research was limited, and this finding is inconsistent with those of some studies [10, 12, 28]. Fujimoto J found that the expression of ERRα was upregulated with tumor progression involving dedifferentiation and myometrial invasion [14]. Our analysis of PPARγ and ERRα expression highlighted differences between benign and malignant tissues. We further confirmed that a significant negative correlation exists between PPARγ expression and ERRα expression in women with EC. Therefore, we believe that crosstalk may occur between PPARγ and ERRα in EC. To the best of our knowledge, this is the first study to analyze the correlation between PPARγ and ERRα in EC patients, and negative correlations between PPARγ and ERRα were observed. Thus, PPARγ may be a protective factor against EC, while ERRα may be an adverse factor for endometrial carcinogenesis. To date, little is known about the crosstalk between PPARγ and ERRα in EC. We found a significant negative correlation between PPARγ and ERRα expression in women with EC. Janice M. Huss et al. found that ERRα activated PPARα gene expression by directly binding to the PPARα gene promoter to control energy metabolism in cardiac and skeletal muscles [29]. PPARs belong to the nuclear receptor (NR) superfamily. Different subtypes of PPARs, including PPARα, PPARβ/δ, and PPARγ, have similar structures. To the best of our knowledge, no studies have proven beyond question that ERRα can activate PPARα gene expression. Wei W et al. found conserved PPREs in the ERRα promoter [13]. When activated by ligand binding, PPARγ forms a heterodimer with another NR, retinoid X receptor (RXR), and then activates gene expression by binding to PPRE. Therefore, PPARγ may inhibit the expression of ERRα by combining with PPRE. Wei W et al. also highlighted a functional link between PPARγ and ERRα pathways, namely, their convergence at the transcriptional coactivator PGC-1β [13]. Through a STRING protein interaction analysis, we found a large number of cofactors shared by PPARγ and ERRα, including PGC-1α, NCOA1, NCOA2, and CREBBP. PGC-1α protein was originally shown to be a coregulator of PPARγ [30], and ERRα has also recently been shown to serve as a functional partner for the PGC-1 family [31, 32], the members of which have emerged as key regulators of mitochondrial metabolism and biogenesis [33, 34]. PGC-1α proteins likely act as surrogate protein ligands for ERRs in regulating the expression of mitochondrial genes and other gene sets involved in maintaining energy homeostasis, as well as a region of retinoic acid receptor α (RARα) [35]. They also activate the activity of a large number of RXR chaperones involved in glucose and lipid metabolism [36]. PPARγ plays important roles in the regulation of several metabolic pathways, including lipid biosynthesis and glucose metabolism. RXR acts as a mandatory partner for PPARγ [35]. PPARγ forms heterodimers with RXR and develops into a complex intact PPARγ-RXRα structure on DNA [37]. Therefore, a tendency toward mutual repression can be speculated to exist between PPARγ and ERRα. PPARγ and ERRα may compete for the same coactivators, including RXR or PGC-1α, to perform their transcriptional functions.

To further explore the possible interaction between PPARγ and ERRα, we also explored the relationship between PPARγ and ERRα in EC cells. Interestingly, when the expression of PPARγ was upregulated by lentiviral-mediated overexpression constructs, the expression of ERRα was decreased. In contrast, when the expression of PPARγ was inhibited by the specific inhibitor GW9662, the expression of ERRα was increased. Moreover, we found that PPARγ expression could also be inhibited by activating ERRα expression. Upregulating ERRα expression could inhibit PPARγ expression in EC cells. After treatment with XCT790, which is an ERRα-specific antagonist [38], PPARγ expression was enhanced in EC cells. Our work is the first to report that PPARγ and ERRα can inhibit one another through a negative feedback loop in EC cells. Our previous study showed that XCT790 can inhibit proliferation and promote apoptosis of EC cells by targeting ERRα [15]. However, we did not discuss the detailed mechanism in our previous work. After data mining based on bioinformatics analysis, we found that the levels of both Bcl2 and Caspase3, which are associated with cellular proliferation and apoptosis, were increased or decreased by PPARγ and ERRα. Furthermore, we confirmed that Bcl2 expression was inhibited, and that Caspase3 expression was promoted after PPARγ activation or ERRα inhibition *in vivo*. Bcl2 expression was promoted, and Caspase3 expression was inhibited after PPARγ was downregulated or after ERRα was upregulated in EC cells. This result was also partly proven by Woo CC et al. [39]. They found that thymoquinone can increase PPARγ activity and downregulate the expression of Bcl2 and Bcl-xL in breast cancer [39]. Weng JR et al. also found that 3β, 7β-dihydroxy-25-methoxycucurbita-5,23-diene-19-al (DMC) can induce PPARγ activation and suppress some PPARγ-targeted signals, including cyclin D1, CDK6, Bcl-2, XIAP, cyclooxygenase-2, NF-κB, and estrogen receptor α, in breast cancer cells [40]. PPARγ activation inhibits Bcl-2 expression, which promotes the mitochondrial release of cytochrome C and initiates apoptosis. Cytochrome C activates Caspase9, which activates downstream Caspase3 and induces apoptosis [41]. Zhang Y et al. found that PGC-1α overexpression in the human epithelial ovarian cancer cell line HO-8910 induced cell apoptosis through coordinated regulation of Bcl-2 and Bax expression in a PPARγ-dependent pathway [42]. Wang M et al. found that PGC-1α can increase the expression of Bcl-2 and promote the survival of mesenchymal stem cells via the PGC-1α/ERRα interaction [43]. Based on the above studies and our experimental results, increasing the expression of PPARγ and decreasing the expression of ERRα can be concluded to have the same effect on downstream signals, such as Bcl2 and Caspase3. PPARγ and ERRα may compete to control these signals by suppressing the function of one another. Apoptosis induced by PPARγ activation or ERRα inhibition still requires further verification. GO and KEGG enrichment bioinformatics analyses indicated that many mutual downstream target genes of PPARγ and ERRα participate in various biological functions, including exocytosis, gene silencing, lipid transport, protein transport, cell proliferation and apoptosis. Whether these two transcription factors compete for the same downstream cofactors requires further study. Simultaneous PPARγ activation and ERRα inhibition may amplify this pro-apoptotic effect and improve the prognosis of EC.

Although most ECs can be detected in the early stage by means of abnormal uterine bleeding after menopause, some patients have unusual patterns of regional and systemic recurrence [44]. Moreover, EC-specific tumor markers are still lacking in clinical practice. Our study confirmed that PPARγ is a protective factor against EC, and that ERRα is a poor prognostic factor for EC in the same population. We further discuss their potential roles as EC tumor biomarkers. Although the ROC curves indicated that ERRα was superior to PPARγ for diagnosing EC, PPARγ was more suitable than ERRα for predicting the survival of EC patients. Both PPARγ and ERRα induce or inhibit the occurrence of EC and showed greater accuracy and effectiveness as markers of EC than other known serum biomarkers, such as CA125 and CA199. However, neither PPARγ nor ERRα alone can serve as an independent risk biomarker to predict the occurrence of EC. Encouragingly, regarding the prediction of EC occurrence, the PPARγ/ERRα ratio is superior not only to PPARγ or ERRα alone but also to currently used clinical serum tumor markers, namely, CA125, CA199, CA153, AFP and CEA. Moreover, surprisingly, the AUC of PPARγ/ERRα for diagnosing EC reached 0.915, with an optimal threshold ratio less than 1.86. In addition, the expression patterns of PPARγ and ERRα may be used to predict the prognosis of patients with EC. Both OS and DFS were lowest for EC patients with PPARγ(-)/ERRα(+) disease. Due to limitations in our study scale, this result should be further confirmed by more studies. However, the combination of PPARγ and ERRα can be used not only to diagnose but also to predict the prognosis of EC.

In conclusion, the expression and immunoreactivity of PPARγ were found to be negatively correlated with the expression and immunoreactivity of ERRα in EC. PPARγ and ERRα compete to control downstream Bcl2/Caspase3 signals to activate or inhibit EC cell proliferation and apoptosis. A dynamic imbalance in PPARγ/ERRα leads to endometrial carcinogenesis. A PPARγ/ERRα ratio≤ 1.86 is an independent risk factor for endometrial carcinogenesis. Moreover, PPARγ(+) EC patients have a favorable prognosis, but PPARγ(-)/ERRα(+) patients have the worst prognosis in terms of both OS and DFS. Simultaneous PPARγ activation and ERRα inhibition may be beneficial in terms of therapeutic efficacy against EC and improve the disease prognosis.

## MATERIALS AND METHODS

### Study population

This study was approved by the ethics committee of Fujian Maternity and Child Health Hospital (FMCH2018-13), Affiliated Hospital of Fujian Medical University. All specimens were collected from 2011.9 to 2017.9, and the patients provided informed consent. The exclusion criteria were as follows: 1. patients with a history of other malignancies; 2. patients with nonepithelial cancers of the uterus, such as carcinosarcoma; 3. patients treated with chemotherapy, radiotherapy or hormone therapy before surgery; 4. patients missing clinical pathology data or with an unclear diagnosis; 5. patients who did not agree to further analysis of their pathological tissue; and 6. patients with a pathological follow-up inconsistent with the original pathological results. The clinical stage of each cancer case was reviewed and diagnosed again according to the FIGO Stage 2014 classification (48 cases of stage I; 13 cases of stage II; 15 cases of stage III; 1 case of stage IV). Thirty cases of well-differentiated endometrioid adenocarcinoma of the endometrium (G1), 27 cases of moderately differentiated endometrioid adenocarcinoma (G2), and 6 cases of poorly differentiated endometrioid adenocarcinoma (G3) were identified. In the same period, 39 samples of endometrial tissue were collected from patients with other benign gynecological diseases. Clinical characteristics, including age, BMI, gravidity, parity, menopause status, and complicated disease cases, including EC with a history of diabetes, hypertension or hyperlipemia, were obtained. Serum tumor markers, including CA125, CA153, CA199, CEA, and AFP, were measured two days before surgery.

### Tissue microarray

Morphological observation to identify typical lesions and markers was performed using hematoxylin and eosin (H&E)-stained sections. Tissue microarray (TMA) sections measuring 1.5 mm in diameter were subsequently dewaxed with xylene/ethanol for immunohistochemical staining.

### Immunohistochemistry

Immunohistochemical staining was carried out on 4-μm-thick sections. The sections were washed with a gradient ethanol/water mixture and then with distilled water. Next, the samples were immersed in citrate buffer (pH 9), boiled for 10 min and treated with 3% hydrogen peroxidase to block endogenous peroxidase activity. A 5% BSA blocking solution was added after antigen retrieval, followed by incubation for 35 min at room temperature. The primary antibody dilutions used in the present study were as follows: 1:25 for PPARγ (ab45036) and 1:100 for ERRα (ab87980) (both from Abcam, Cambridge, MA, USA). Primary antibody incubation was performed overnight at 4°C in a humidified chamber. The results were evaluated with a semiquantitative integration method and scored according to the staining range and staining intensity. The slides were evaluated by 2 of the authors who were blinded to the patient characteristics and outcomes using a standard light microscope. A semiquantitative grading system incorporating staining intensity (score, 0–3) and tumor area with positive staining (0, 0%; 1, 1%-25%; 2, 26%–50%; 3, 51%-75%; and 4, 76%-100% of tumor cells) was applied. The staining index (SI) was calculated as the product of staining intensity and area and ranged from 0 to 12, as described in several publications [45–47]. Immunohistochemical activity was stratified by the SI (-, 1-2; +, 3-4; ++, 5-8; and +++, 9-12). Immunological activities were classified as positive (+, ++, +++) and negative (-).

### Cell culture

The human endometrial carcinoma cell lines RL-952, ECC-1, HEC-1A, and HEC-1B were purchased from the American Type Culture Collection (ATCC) and maintained in our laboratory. All cell lines were identified by BIOWNG Biotechnology, Shanghai, China. All cells used were the most common *in vitro* EC cell models. The RL-952 and ECC-1 cell lines were positive for ERα expression, but the HEC-1A and HEC-1B cell lines were negative for ERα expression. These model differences created conditions for exploring the relevance of PPARγ and ERRα. RL-952 cells were cultured in DMEM/F12 medium supplemented with 0.005 mg/mL insulin and 10% fetal bovine serum (FBS), and ECC-1 cells were cultured in F12 medium supplemented with 10% FBS. HEC-1A and HEC-1B cells were cultured in high-glucose DMEM supplemented with 10% FBS at 37°C in a 5% CO2 atmosphere. All culture media were purchased from Thermo Fisher Scientific.

### Quantitative real-time polymerase chain reaction (qRT-PCR)

All RNA was extracted according to the TRIzol reagent protocol (Invitrogen, Carlsbad, CA, USA). Briefly, 1 μg of DNase I-treated RNA was reverse transcribed into complementary DNA (cDNA) using a reverse transcription kit (Promega). The PCR primer sets used were as follows: ERRα, sense 5′-ACC GAG AGA TTG TGG TCA CCA-3′, antisense 5′-CAT CCA CAC GCT CTG CAG TACT-3′ (101 bp); glyceraldehyde 3-phosphate dehydrogenase (GADPH, control), sense 5′-GCA CCG TCA AGG CTG AGA AC-3′, antisense 5′-TGG TGA AGA CGC CAG TGGA-3′ (138 bp); and PPARγ, sense 5′-AATGGAAGACCACTCCCACT-3′, antisense 5′-GGTACTCTTGAAGTTTCAGGTC-3′ (152 bp). Experiments were carried out in triplicate and repeated twice. The relative level of ERRα mRNA was quantified by the image-intensity ratio of the target gene mRNA to GAPDH mRNA. The target gene mRNA level was quantified using a comparative method (2^-ΔΔCT^ method) and normalized to GAPDH expression.

### Western blot analysis

Cell lysates were prepared with a lysis/extraction reagent (Sigma). Protein concentrations were determined using a bicinchoninic acid (BCA) protein assay reagent kit (Pierce). Protein samples (30 μg) were resolved by 12% SDS-polyacrylamide gel electrophoresis, blotted onto PVDF membranes and blocked with TBS-Tween 0.1% containing 5% nonfat dry milk at room temperature for 1 h. Blotted membranes were incubated with anti-human PPARγ rabbit monoclonal antibody (1:500 dilution, Abcam, Cambridge, MA, USA) or anti-human ERRα rabbit monoclonal antibody (1:300 dilution, Abcam) overnight at 4°C. β-Actin rabbit monoclonal antibody (1:1000 dilution; Beyotime, Shanghai, China) was used as the control. An enhanced chemiluminescence (ECL) detection system (Thermo) was used to visualize the bands.

### Lentivirus-mediated vector construction and infection

Full-length cDNA plasmids for PPARγ (PPARG, NM_015869) and ERRα (ESRRA, NM_004451) were purchased from Genechem (Shanghai, People's Republic of China) and cloned into a lentivirus-based vector carrying the green fluorescent protein (GFP) gene (GV358, Genechem, Shanghai, China) with the following component sequence: Ubi-MCS-3FLAG-SV40-EGFP-IRES-puromycin. EC cell lines transfected with the lentiviral vector were used as negative control (NC) groups, and cell lines without lentivirus treatment were used as blank controls. The lentiviral vector constructs carrying PPARγ, ERRα and NC were used to infect EC cells at a multiplicity of infection (MOI) of 100. After 72 h of infection, GFP expression was detected to calculate the infection efficiency. Cells were harvested at an infection rate >90%.

### Data mining based on bioinformatics analysis

The expression of PPARγ and ERRα in EC was determined using the TCGA database from UALCAN (http://ualcan.path.uab.edu/). The interaction between PPARγ and ERRα was analyzed based on the search tool for the retrieval of interacting genes/proteins (STRING, https://string-db.org/cgi/input.pl) database. Downstream target genes common to PPARγ and ERRα were screened in the Gene Transcription Regulation Database (http://gtrd.biouml.org/). The ModFit of PPARγ and ERRα was based on the JASPAR database, which provides the most comprehensive public data on transcription factors and DNA binding site motifs (http://jaspar.binf.ku.dk/). The selected genes were subjected to gene function annotation based on the GO database to analyze the significant functions exhibited by the coexpressed genes. Pathway analysis was based on the KEGG database. GO and KEGG analyses were performed using the Database for Annotation, Visualization and Integrated Discovery (DAVID, https://david.ncifcrf.gov/home.jsp). Significant screening criteria were set at P < 0.05.

### Drug treatment and cell proliferation assay

Cells were cultured in 96-well plates, and XCT790 and GW9662 purchased from Sigma (Sigma-Aldrich, St. Louis, MO, USA) were used for the experiments. XCT790 is a specific inhibitor of ERRα, and GW9662 is a PPARγ antagonist. Previous studies have found that the optimal concentration of XCT790 in EC cells is 10 μM. Through preliminary experiments, the optimal drug concentration of GW9662 was determined to be 5 μM. XCT790 and GW9662 were dissolved in dimethyl sulfoxide (DMSO) according to the manufacturer’s instructions. Drug-treated cells were used as the experimental groups, drug-free DMSO-treated cells were used as the NC groups, and untreated cells were used as the blank control groups. The cells in each group were treated with 10 μl of CCK-8 solution (MedChemExpress, China) for 0 h, 24 h, 48 h, 72 h, and 96 h, and after 1 h in an incubator, the absorbance values at 450 nm were measured by a microplate reader.

### Apoptosis analysis

For flow cytometric analysis, all cells treated with GW9662, XCT790 or lentivirus were seeded into 6-well plates. When the cells reached 80% confluence, trypsin with no EDTA was added, and the cells were harvested. After centrifugation, the cell pellets were washed twice with precooled phosphate-buffered saline (PBS). Cells were resuspended in buffer at 10^6^ cells/mL. Then, cells were stained with an Annexin-V-FLUOS or 7-AAD staining kit (BD, USA) according to the manufacturer’s instructions. Apoptotic cells were analyzed using the FACS Canto II flow cytometer (BD, USA) and Diva software (BD, USA). All experiments were performed in triplicate. Data were analyzed using the ModFit LT (Verity Software, Topsham, ME, USA) and Cell Quest (BD Biosciences) software packages.

### Statistical analysis

Statistical analyses were performed using SPSS 17.0 or MedCalc software for Windows. A P value less than 0.05 was considered statistically significant. The results were evaluated using the T-test, Mann-Whitney U test, or Wilcoxon test. Categories were compared using the Pearson χ^2^ or Fisher’s exact test where appropriate. ROC curves were used to evaluate the diagnostic value of PPARγ and ERRα mRNA expression. A logistic model was used to determine the risk of EC. Univariate survival analyses of the time to death due to endometrial carcinoma (OS and DFS) were conducted using the Kaplan-Meier method. The entry date was the date of primary surgery. Patients who died from other causes were censored at their date of death. Differences in survival between groups were estimated by 2-sided log-rank (Mantel-Cox) tests. The results were considered significant when P < 0.05.
